# T, B, and NKT Cells in Systemic Inflammation in Obstructive Sleep Apnoea

**DOI:** 10.1155/2015/161579

**Published:** 2015-05-25

**Authors:** Joanna Domagała-Kulawik, Iwona Osińska, Aleksandra Piechuta, Piotr Bielicki, Tomasz Skirecki

**Affiliations:** ^1^Department of Pneumonology and Allergology, Medical University of Warsaw, Ulica Banacha 1a, 02 097 Warsaw, Poland; ^2^Laboratory of Flow Cytometry, Medical Centre of Postgraduate Education, Ulica Marymoncka 99, 01 813 Warsaw, Poland

## Abstract

*Background*. Obstructive sleep apnoea syndrome (OSAS) brings risk of serious complications. The study objective was to assess elements of the cellular immune response in the course of OSAS. *Methods*. Peripheral blood (PB) lymphocytes: T, B, NK, NKT-like, Th, Tc, and HLA DR+ T cells were evaluated by flow cytometry of 48 OSA patients; the concentration of adiponectin, interleukin 1*β*, and TNF*α* was measured by ELISA method. The *OSA complication score* was developed and used for statistical analysis. *Results*. The proportion of B cells and Th/Tc ratio were significantly lower in the BP of OSA patients when compared with control subjects (median 7.9 versus 10.9%, 0.9 versus 1.5, *p* < 0.05). The proportion of Tc, NK, NKT-like, and HLADR positive T cells were elevated in OSA patients when compared with healthy subjects (36.4 versus 26.8, 15.5 versus 8.5, 5.7 versus 3.0, and 8.4 versus 4.5%, *p* < 0.05, resp.) and were more pronounced in patients with metabolic syndrome. The grade of *OSA complication score* correlated with systemic inflammation markers and the proportion of B cells. The value of adiponectin/BMI ratio correlated significantly with SpO_2_ (*r* = 0.31, *p* < 0.05), CRP (*r* = −0.35, *p* < 0.05), TNF*α* concentration (*r* = −0.36, *p* < 0.05), and proportion of B cells (*r* = 0.32, *p* < 0.05). *Conclusion*. Lymphocytes B, Tc, NK, NKT-like, and adiponectin are involved in systemic immune response in OSA patients possibly predisposing them to cardiovascular and metabolic complications.

## 1. Introduction

Obstructive sleep apnoea syndrome (OSAS) is the most frequent clinical form of breathing disorders during sleep in patients who come to the sleep medicine specialist. According to the epidemiological research conducted in Poland, the prevalence of the disease is estimated as 8.7% in men and 2.5% in women aged 40–72 yrs. [1]. The most important risk factors for OSAS are obesity, craniofacial structural abnormalities (curvature of the nasal septum, hyperglossia, structural abnormalities and position of the mandible, overgrown tonsils, and structural abnormalities of the soft palate and the uvula), and a decreased upper airway muscle activity. Breathing disorders during sleep increase the risk of cardiovascular complications and especially risk of arterial hypertension [2, 3]. The most pronounced complication and/or coexisting disorder with OSAS is the metabolic syndrome [3]. The OSAS and related comorbidities are accompanied by systemic inflammation with involvement of well-known inflammatory mediators: C-reactive protein (CRP), coagulation factors, lipids, and carbohydrates [2, 4–6]. The participation of proinflammatory cytokines interleukin 1*β* (IL-1*β*) and tumor necrosis factor *α* (TNF*α*) in OSA systemic inflammation was established in lots of studies [2, 4, 7, 8]. Furthermore a reduced concentration of adiponectin, the cytokine of anti-inflammatory properties, was observed [4, 9, 10]. Up to now, some selective data on the influence of OSA on the alterations of lymphocyte population in systemic circulation exist [11–13]. The aim of our study was to investigate the profile of the main lymphocyte subsets in the peripheral blood of patients with severe OSAS with regard to the indices of systemic inflammation.

## 2. Materials and Methods

### 2.1. Subjects and PSG

48 patients with confirmed OSAS were investigated. The diagnosis of OSAS was established in accordance with the American Academy of Sleep Medicine (AASM) and Polish Respiratory Society recommendations [14, 15]. All patients underwent a 7-hour polysomnography during which we monitored airflow, chest and abdominal motion, and the oxygen arterial blood saturation. A polysomnography test (PSG) was executed with the help of Alice 4 apparatus from RESPIRONICS (USA). Airflow was measured with the use of a pressure cannula or a three-channel thermistor placed in the front nostrils and the mouth area. Chest and abdominal motion were recorded with the use of two piezoelectric belts (around abdomen and chest). Peripheral capillary oxygen saturation (SpO_2_) was recorded with a pulsoximeter. The electroencephalogram (EEG), electrooculogram (EOG), and electromyogram (EMG) were recorded to determine sleep structure and number of awakenings. During sleep cardiac function was monitored by a one-lead ECG. The Epworth Sleepiness Scale (ESS) was used to assess the daytime sleepiness [16]. The diagnosis of OSAS was made when the AHI value exceeded 5 and over 10 points in the ESS.

### 2.2. Demographics and Laboratory Tests

The basic clinical data were obtained from all the participants, including age, ethnicity, vital signs, weight and BMI, occupation, and a history of smoking status. The following comorbidities were taken into consideration: hypertension, ischemic heart disease, diabetes, stroke, and chronic obstructive pulmonary disease (COPD). In each patient fasting complete blood count, hemoglobin concentration (Hb), plasma glucose (FPG), total cholesterol (TC), triglycerides (TG), high density lipoprotein cholesterol (HDL-C), and CRP were analyzed in the Central Laboratory of the University Hospital.

**Figure 1:**
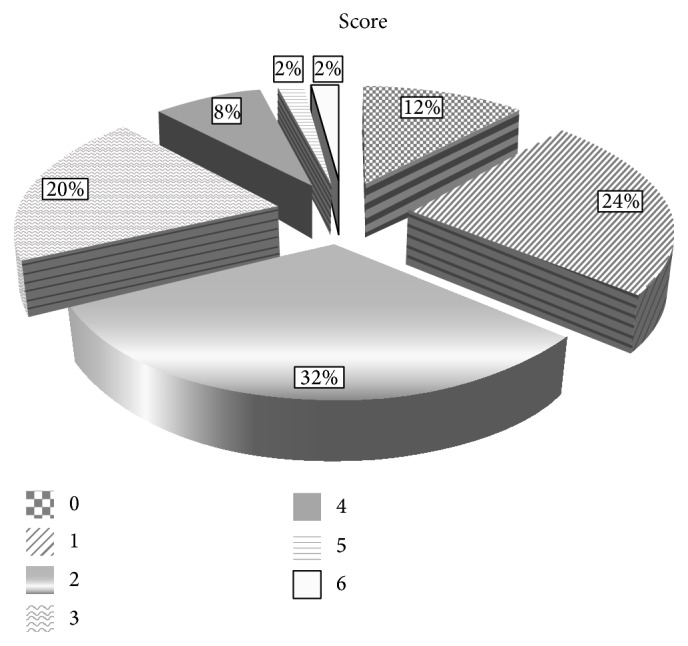
*OSA complication score* and the number of patients distribution in each group. The description of* score* was included in the main text.

For the analysis of clinical data we divided patients to those with and without obesity as well as with and without metabolic syndrome. The obesity was established by body mass index (BMI) as high as 30 kg/m^2^ or higher. The metabolic syndrome was defined according to the International Diabetes Federation (IDF) by obesity and a presence of at least two of four features: low HDL concentration (<40 mg/dL in men and <50 mg/dL in women), high triglycerides concentration (>150 mg/dL), hypertension, or diabetes [17]. In addition we designed the authors' own* OSA complication score* by adding up the following records: obesity, diabetes, hypertension, ischemic heart disease, stroke, and current smoking history. The distribution of patients number in each group of score from 1 to 6 is presented in Figure 1. The demographic data of the patient group are collected in Table 1.

The control group consisted of 20 healthy volunteers without any chronic disease, who did not receive any treatment, both sexes (12 women, 8 men), in the mean age 46 (30–76 years). 5% smokers constituted 25% of this group; the mean BMI was 24.32 ± 3.01 kg/m^2^. The study was approved by the Ethics Committee of the Medical University of Warsaw and all the participants gave informed consent.

### 2.3. Flow Cytometry

The venous blood samples were collected before breakfast, early morning. All the analyses were performed right after blood collection. We analysed the proportion of the following lymphocyte subtypes: T cells, B cells, T helper (Th) and T cytotoxic cells (Tc), natural killer cells (NK), natural killer T cells (NKT-like), and T cells with HLA-DR expression by Simultest (Becton-Dickinson Immunocytometry Systems, San Jose, California). The following mixtures of antibodies were used: CD3-FITC/CD19-PE, CD4-FITC/CD8-PE, CD3-FITC/Anti-HLA-DR-PE, and CD3-FITC/CD16+CD56-PE. In the flow cytometry analysis, first, anti-CD45-FITC and anti-CD14-PE were used for the lymphocyte gate setting at FSC/SSC graph. As a negative isotype control the IgG_1_-FITC/IgG_2a_-PE were applied. The analyses were performed using flow cytometry method (FACS Canto II flow cytometer, Becton-Dickinson, San Jose, California), the cells being collected by Diva software (BD). The analysis was performed in the same manner, with the same set of antibodies and in the same conditions in patients and control group. The white blood count was measured in automatic hemocytometer. Total cell number of a particular lymphocyte subpopulation was calculated from WBC and frequency of given population in flow cytometry analysis, next reported as number of cells per *μ*L.

### 2.4. Cytokine Measurements

The serum concentration of adiponectin was measured using Human Total Adiponectin/Acrp30 Immunoassay kit (R&D System, USA) and ELISA method according to the prescription by the producer. For TNF*α* and IL-1*β* concentration measurement we used Human TNF-*α* and IL-1*β* Immunoassay, respectively (R&D System, USA).

### 2.5. Statistical Analysis

For data comparison the Mann-Whitney *U* test and Kruskal-Wallis test (for data nonnormally distributed) were applied. *p* < 0.05 was regarded as significant. The relationships between the data were examined by Spearman's rank correlation coefficient. Correlations with both *r* ≥ 0.3 and *p* < 0.05 were considered relevant.

## 3. Results

In Table 2 we present the results of flow cytometric analysis of lymphocyte populations in the studied groups and the phenotype of each cell population is shown. The analyses revealed significantly lower proportion and the number of B cells in the PB of patients with OSAS when compared with control subjects, so were the proportion and a number of Th cells. The Th/Tc ratio was significantly lower in patients than in healthy subjects (median value 0.9 versus 1.5, *p* = 0.003). The proportions and the numbers of Tc, NK, NKT-like, and HLA-DR positive T cells were elevated in OSA patients when compared with healthy subjects: the values and statistical significances are presented in Table 2. These differences were pronounced in obese patients and in patients with metabolic syndrome (Table 3).

**Figure 2:**
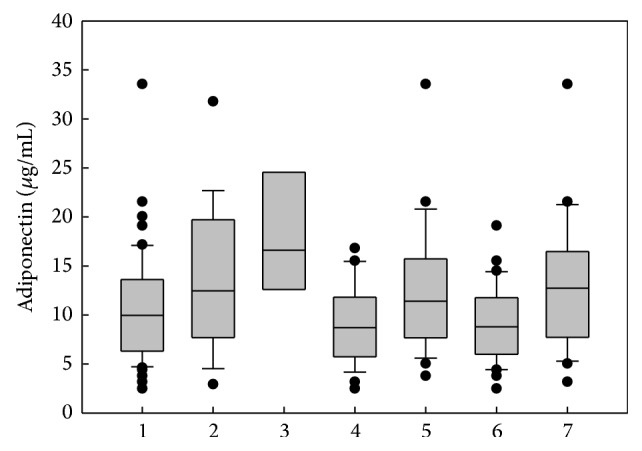
Median concentration of adiponectin in peripheral blood of OSA patients and healthy subjects. Plots are the 10th, 25th, 75th, and 90th percentiles as vertical boxes with error bars. 1: all OSA patients; 2: healthy subjects; 3: OSA patients with BMI < 25; 4: obese OSA patients; 5: no obese OSA patients; 6: OSA patients with metabolic syndrome; 7: OSA patients without metabolic syndrome. There was significant difference between groups 3 and 4, 6, and 7 in Kruskal-Wallis test (*p* < 0.05).

The median serum concentration of adiponectin was significantly reduced in OSA patients when compared with healthy subjects (*p* < 0.05) and was lowest in obese OSA patients (Figure 2). Similarly, the index, adiponectin to body mass index (A/BMI), differed significantly between patients with OSA and healthy subjects: median value 0.30 (0.19–0.44) versus 0.62 (0.41–0.99), respectively, *p* = 0.0006. The median A/BMI index was lowest in obese OSA patients and was 0.26 (0.17–0.36). It was also decreased in nonobese OSA patients and differed when compared with healthy subjects: median value 0.42 (0.27–0.30) versus 0.62 (0.41–0.99), respectively, *p* = 0.08.

The median serum concentration of IL-1*β* was 0.30 pg/mL (0.02–0.49) and the concentration of TNF*α* was 0.90 pg/mL (0.57–1.28) in the OSA group. Levels of these cytokines did not differ between patients and healthy control subjects. The median concentration of IL-1*β* and TNF*α* in patients with metabolic syndrome was elevated when compared with patients without metabolic alterations. It was, respectively, 0.34 versus 0.21 pg/mL for IL-1*β* and 0.97 versus 0.82 pg/mL for TNF *α* (differences not significant).

When we analysed the influence of coexisting disorders on immune alterations in OSA patients we found a strong relation of obesity and metabolic syndrome therewith. The significant relations of immune parameters with obesity and BMI are presented in Table 3. In patients with coexisting COPD the proportion of B cells was significantly lower when compared with OSA patients without COPD (3.4 (1.6–5.1) versus 8.5 (6.0–11.0), *p* = 0.005). No relevant influence of tobacco smoking on investigated immune markers was found.

There was a significant adverse relation of B cells proportion with CRP concentration (*r* = −0.28, *p* < 0.05).

The value of A/BMI ratio correlated significantly with SpO_2_ (*r* = 0.31), with CRP (*r* = −0.35), and with proportion of B cells (*r* = 0.32) in the whole patients group, while it correlated with TNF*α* concentration (*r* = −0.36) only in the group of obese patients. In nonobese OSA patients group there was significant adverse relation of A/BMI ratio with the proportion of Th cells (*r* = −0.44). We noticed the tendency of inversed correlation between A/BMI and AHI.

**Figure 3:**
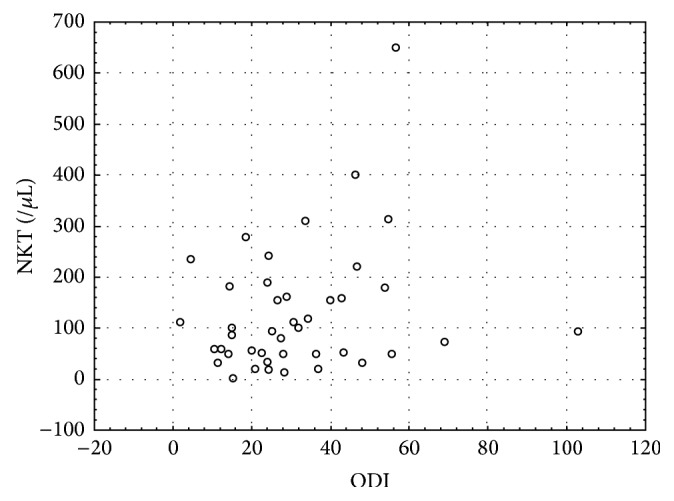
Significant correlation between oxygen desaturation index (ODI) and the number of NKT-like cells in the peripheral blood of OSA patients (*r* = 0.4, *p* < 0.05).

Of the indices of OSA severity we found the relation of ODI with lymphocyte subtypes only in patients burdened with obesity: a positive correlation of ODI with proportion and a number of NKT-like cells was observed (*r* = 0.40, *p* < 0.05), presented in Figure 3.

In Table 4 we present the results of the correlation test of OSA severity indices, proportion of lymphocyte subtypes, and the level of inflammatory mediators with BMI and the grade of* OSA complication score*.

We did not find more relevant correlations between proportions of cell subtypes and the concentrations of adiponectin, IL-1*β*, and TNF*α* with the demographic data. The mean age in the patients group was significantly higher than in the control group. However, after matching investigated individuals into the groups of comparable age, the differences in the inflammatory indices noted above remained significant.

## 4. Discussion

There are many records supporting the inflammatory nature of OSAS-related comorbidities and the participation of inflammatory cytokines in this process. However, little is known about a character of the cellular immune response in this syndrome. Thus the aim of the present study was the analysis of lymphocyte subtypes in peripheral blood of OSA with regard to the main subpopulations. We observed significant influence of OSAS and OSAS complications on the amounts of circulating inflammatory cells, lymphocytes B, Tc, NK, and NKT-like cells, and on the concentration of adiponectin. The studied group consisted of patients with severe OSA. We suspect that the elements of immune alterations observed by us are a consequence of OSA* per se*.

In our previous studies on local and systemic inflammation in different lung diseases we observed that the changes of lymphocytes profile belong to the most stable and repeatable characteristics of immune response [18–20]. The defined profile of immune system constituents seems to reveal the balance between their activation and suppression and between induction of immune response and the resolution and repair. Usually, patients suffer from OSA for many years before diagnosis and the long-time developing adaptive process should be taken into account. Finally, the severity of the disease varies: in some individuals the hypoxia is deeper while in others it is of slighter degree; thus the level of activation of immune response and anti-inflammatory protection is highly individual. It seems probable that most of immune alterations in the course of OSA are fluent and we detected those changes which are definitely persistent.

The most pronounced disturbances of the cellular response and adiponectin concentration were observed in patients with obesity, metabolic syndrome, and high* OSA complication score* which is in agreement with other observations [2, 3, 6, 10]. We are willing to suspect the following sequence: intermitted hypoxia-inflammation-clinical complications. Chronic intermitted hypoxia acts on the molecular level by activation of the nuclear factor *κ*B (NF*κ*B) as well as hypoxia inducible factor 1 (HIF1-*α*) and triggers production of inflammatory mediators by oxidative stress. These mediators are also known to affect vascular endothelium [4, 5, 21].

In the studies of Dyugovskaya et al. on cellular immune response in OSAS, T lymphocytes presented high expression of markers of activation and cytotoxicity, which are AHI dependant. The most affected subtypes were CD8+ cells [12]. The proportion of CD8+ cells bearing CD16 and CD56 NK markers were significantly higher in OSA. It was accompanied by enhanced cytotoxic properties of CD8+ cells. The treatment with nCPAP contributed to normalization of the expression of these markers on CD8 cells. The authors concluded that the presence of CD8+/CD56+/perforin positive cells is involved in inflammatory process leading to cardiovascular complications of OSA [12]. Tan et al. also confirmed high proportion of CD8+ cells and low proportion of CD4+ cells. These changes were AHI dependent in children suffering from OSA [22]. The results of our study: the imbalance of Th/Tc with high Tc (CD8+) proportion remains in agreement with what was presented above and with other Dyugovskaya and Lavie results [13]. Interestingly, we observed some alteration of NK cells (the innate lymphoid CD3-negative cells bearing CD56). We also observed an augmentation of NKT cells (T cells having both NK and T characteristics, CD3+/CD56+). NKT cells link NK and T cell population bearing similar markers and playing similar immunoregulatory function [23]. There are two members of NKT family in humans: invariant-NKT (iNKT) cells which are CD1d stimulated and NKT-like cells which are MHC dependant for antigen presentation [24]. After antigen stimulation NKT cells release Th1, Th2, and Th17 cytokines [24]. The augmentation and increased activity of lung NKT-like cells were described in COPD [25]. In the experimental model of COPD, the iNKT cells responded to cigarette smoke-induced oxidative stress [26]. Oxidative stress is an inherent part of OSAS pathophysiology and the similar NKT activation process could be taking place in OSAS and COPD. The function of the iNKT cells is related to CD1d antigen-presenting molecule recognized lipids [27]. During normal homeostasis there are a high number of iNKT cells in the adipose tissue, while in the obesity their proportion is disturbed and these cells possess increased capacity of production of the proinflammatory cytokines [28]. NKT cells and especially iNKT cells were also shown to contribute to the pathophysiology of atherosclerosis, a well-known complication of OSAS [29, 30]. Unfortunately, we investigated NKT cells without precise discrimination to iNKT which needs further investigation in OSAS.

The well-known function of lymphocytes B is humoral immunity but there is growing body of evidence that B cells play also very important role in suppression and regulation of immune response. It is comprehended by release of interleukin 10 (IL-10) and by expression of programmed death ligands 1,2 (PD-L1, PD-L2), Fas-L, and granzyme B which induce T cells apoptosis [31]. Recently, the functional disturbances of B cells in adipose tissue were described [32]. We observed for the first time a decreased number of B cells in the PB of OSA patients with strong relation to metabolic disorder and obesity. In our study the proportion of B cells was very low in patients with the overlap syndrome OSAS/COPD, but the role of B cells in COPD was highlighted by others [33]. Thus the depletion of circulating B cells seems to promote systemic inflammation in OSAS. Furthermore regulatory B cells present interaction with NKT by CD1d molecule [31]. Our results showing low proportion and number of B cells and augmented population of NKT-like cells in OSA patients are agreeable with the newest data.

We attempted to assess the participation of selected cytokines in the OSA-induced inflammatory process; however, we did not obtain any distinct result. The results of the analysis of cytokines concentration should be interpreted with caution; there are many factors influencing these indices. Serum cytokine level is dynamic and it depends on many conditions: for example, rapid increase of TNF*α* was observed just after apnoeic events; the IL-6 is secreted after effort [4, 34, 35], although we confirmed the value of the detection of adiponectin concentration in the blood of OSA patients. Adiponectin (known also as adipocyte complement-related protein of 30 kDa, Acrp30) is secreted by adipocytes and regulates metabolic processes. The normal range is 2–10 *μ*L/mL and is stable [36]. In adults the adiponectin concentration is inversely proportional to body mass [21]. The role of adiponectin in regulation of immune system was noted, among others, by antagonizing TNF*α*, IL-6 secretion and function, induction of IL-10 production, polarization of Th cells to T regulatory population, and polarization of macrophages from M1 to M2 cells [4, 10, 37]. In our study, the adverse significant correlation of adiponectin concentration with TNF*α* level and CRP was noted. We confirmed an adverse correlation of adiponectin concentration with body mass index of OSA patients which was previously described in many studies [3]. It was presented that nCPAP treatment influenced adiponectin concentration only in obese patients which suggests that not OSA but obesity affects adiponectin pathway. We found the relation of A/BMI with our original* OSA complication score*: the lower the A/BMI index, the higher the risk of cardiovascular and metabolic complications of OSA. We observed low concentration of this cytokine also in nonobese OSA patients and a correlation of this cytokine concentration with important index of OSA severity, that is, SpO_2 _min. Al Mutairi et al. also confirmed that the adiponectin level may serve as a marker of OSA severity [9]. Available data are conflicting: in one study no influence of comorbidities on adiponectin level was found [8]; Kosacka et al. in their study on the large group of patients concluded that OSAS does not influence adiponectin level but diabetes was complicated by low level of this cytokine [38]. But, the results of our study emphasised the role of adiponectin in the immunity of OSA* per se* with possible protective role against development of OSA complications [3, 37].

In conclusion, we present for the first time the participation of lymphocytes B, NK, and NKT-like cells in the systemic inflammation in the course of OSAS. These changes are stronger in patients with OSA complications. In addition, the involvement of adiponectin in immune response in OSAS was confirmed. Moreover, the newly developed* OSA complication score* was proved useful for assessment of common OSA-induced comorbidities and may become a useful tool in further studies.

## Figures and Tables

**Table 1:** Demographic data, results of polysomnographic tests and metabolic characteristic of patients with OSA (data expressed as mean ± SD).

Patients number	48

Age (years, mean, [range])	58.5 [36–82]

Sex (female/male)	15 : 33

Smokers (*n*,%)	*n* = 17 35%

Mean pack years	5.80 ± 10.54

BMI (kg/m^2^)	31.68 ± 6.75

Obesity (%)	*n* = 27 56%

AHI (h^−1^)	52.92 ± 21.02 (8–110)

sAHI (%)	*N* = 44 92%

ODI	31.11 ± 18.51

SpO_2_ Lowest Mean	76.06 ± 8.40% 90.88 ± 3.20%

ESS	12.23 ± 5.23 (2–21)

WBC (×10^3^/*μ*L)	7.08 ± 1.78

Hb (g/L)	14.51 ± 1.34

CRP (mg/L)	4.35 ± 6.45

Total cholesterol (mg/dL)	196.02 ± 38.73

HDL-C (mg/dL)	50.10 ± 14.03

TG (mg/dL)	160.4 ± 124.03

Hypertension (%)	*n* = 34 71%

Diabetes (%)	*N* = 8 17%

Ischemic heart disease (%)	*n* = 10 21%

COPD (%)	*N* = 4 8%

Metabolic syndrome	*N* = 27 56%

AHI: apnea/hypopnea index; sAHI: severe OSA; ODI: oxygen desaturation index; ESS: Epworth Sleepiness Scale; SpO_2_: peripheral capillary oxygen saturation; HDL-C: high density lipoprotein cholesterol; TG: triglycerides; COPD: chronic obstructive pulmonary disease.

**Table 2:** Proportion of lymphocyte subtypes and CD4+ : CD8+ ratio in the peripheral blood of patients with OSA and healthy subjects. Data expressed as median (p25–p75).

Lymphocyte subset (%)	OSA (*n* = 48)	Healthy subjects (*n* = 20)	*p*
B CD19+	7.9 5.6–10.7	10.9 8.5–13.4	<0.05

T CD 3+	74.4 67.0–80.0	71.7 67.8–77.2	NS

Th CD4+	35.5 30.9–44.4	42.4 39.7–49.7	0.005

Tc CD8+	36.4 28.1–45.1	26.8 23.1–38.7	0.02

Th : Tc	0.9 0.6–1.5	1.5 1.2–1.9	0.0003

NK CD3−CD16+/CD56+	15.5 11.5–24.3	8.5 6.9–14.6	<0.05

NKT-like CD3+CD16+/CD56+	5.7 2.8–11.2	3.0 1.8–5.8	<0.05

CD3+/HLA DR+	8.4 5.1–12.2	4.5 3.8–6.5	<0.05

**Table 3:** Presentation of significant differences of OSAS indices, inflammatory mediators, and selected lymphocyte subpopulations between patients in relation to obesity and metabolic syndrome.

		Obesity (*n* = 27)	Nonobese (*n* = 21)	*p*	MS (*n* = 26)	Without MS (*n* = 22)	*p*
AHI	h^−1^	62.7 39.4–71.6	42.4 34.0–51.3	0.002	46 39–72	46 34–63	NS

SpO_2_ min	%	74.0 67.0–79.0	81.0 78.5–85.5	0.0002	75 68–80	80 77–82	0.038

CRP	mg/dL	3.6 1.4–7.1	1.0 0.6–2.2	0.002	3.6 1.6–5.5	1 0.6–2.1	0.002

WBC	×10^3^/*μ*L	7.4 6.0–9.1	6.5 5.6–7.2	0.04	6.9 6–8.9	6.7 5.3–7.4	NS

Lymphocytes B	%	6.5 5.4–8.5	10.5 6.4–12.4	0.005	7.0 5.4–10	9.6 6.7–12.4	NS

NK cells	*n*/*μ*L	333 244–474	209 126–294	0.006	329 209–474	258 149–337	0.03

NKT-like cells	*n*/*μ*L	111 58–195	55 32–123	0.02	103 52–183	88 35–179	NS

CD3+/HLA DR+	*n*/*μ*L	149 85–242	113 55–158	0.03	162 81–245	113 68–153	NS

AHI: apnea/hypopnea index; SpO_2_: peripheral capillary oxygen saturation; WBC: white blood cells; MS: metabolic syndrome.

**Table 4:** Correlation of the indices of OSA severity, inflammatory markers, and selected lymphocyte subtypes with the index of *OSA complication score* and BMI (expressed as *r* > 0.3, significant *p* < 0.05).

	Score	BMI	A/BMI
AHI	0.32	NS	NS
SpO_2_ min	−0.38	NS	0.32
SpO_2_ mean	−0.41	NS	NS
CRP	0.44	0.54	−0.35
Fibrinogen	0.39	0.32	NS
WBC	0.51	0.36	NS
B cells%	−0.31	−0.53	0.34
NK%	NS	0.35	NS
CD3+/HLADR+%	NS	0.3	NS
TNFα	NS	NS	−0.36
A/BMI	−0.4	—	—
Adiponectin	NS	−0.36	—

AHI: apnea/hypopnea index; SpO_2_: peripheral capillary oxygen saturation; WBC: white blood cells; BMI: body mass index; A: adiponectin.
